# Supplementary material for: Physiological and morphological correlates of blood parasite infection in urban and non-urban house sparrow populations
Source: PLoS One. 2020 Aug 19;15(8):e0237170. doi: 10.1371/journal.pone.0237170 (PMC7437892; doi:10.1371/journal.pone.0237170)
Supplement: S1 Fig — (DOCX) [file pone.0237170.s001.docx]

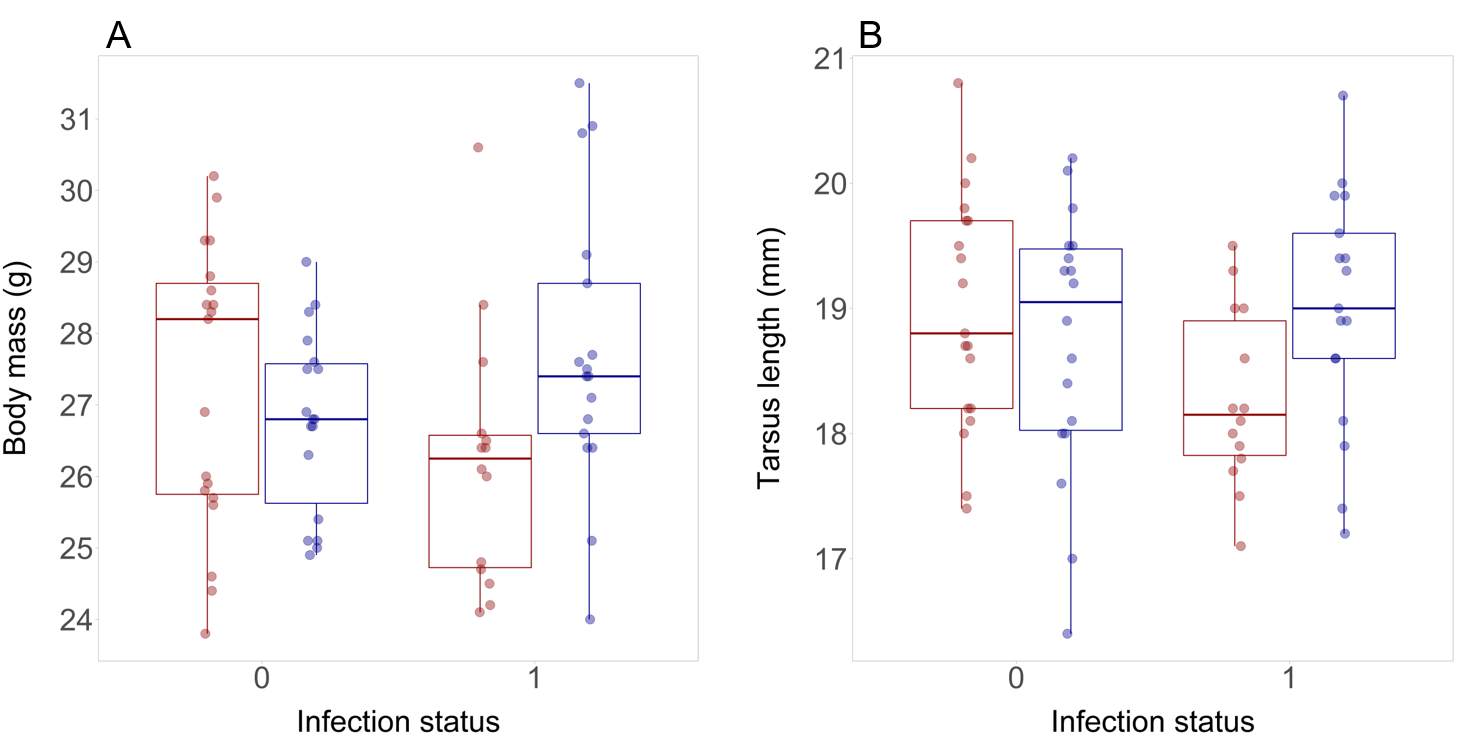


S1 Fig. (A) Body mass and (B) tarsus length in relation to infection status (0 = uninfected, 1 = infected) and sex in adult house sparrows. Dots represent the raw data. Red boxplots and points correspond to the females, and blue boxplots and points to the males.
