# Supplementary material for: Physiological and morphological correlates of blood parasite infection in urban and non-urban house sparrow populations
Source: PLoS One. 2020 Aug 19;15(8):e0237170. doi: 10.1371/journal.pone.0237170 (PMC7437892; doi:10.1371/journal.pone.0237170)
Supplement: S2 Fig — (DOCX) [file pone.0237170.s002.docx]

S2 Fig. Body condition in relation to population, urbanisation score and infection status in adult house sparrows. Dots represent the raw data. Black and orange boxplots and points represent the uninfected and the infected birds, respectively.
