# Supplementary material for: Physiological and morphological correlates of blood parasite infection in urban and non-urban house sparrow populations
Source: PLoS One. 2020 Aug 19;15(8):e0237170. doi: 10.1371/journal.pone.0237170 (PMC7437892; doi:10.1371/journal.pone.0237170)
Supplement: Attachment — Submitted filename: PONE-D-20-04379R1 review.docx [file pone.0237170.s005.docx]

Manuscript	[Click here to access/download;Manuscript;MS_malaria- physio_150420.docx](https://www.editorialmanager.com/pone/download.aspx?id=26411076&amp;guid=dbc05c8f-294f-47e0-ac1b-351fc3b55405&amp;scheme=1)

## 1 Physiological and morphological correlates of blood parasite infection in urban and non-

2 **urban house sparrow populations**
3 Coraline Bichet^1^, François Brischoux^2^, Cécile Ribout^2^, Charline Parenteau^2^, Alizée Meillère^2^
4 and Frédéric Angelier^2*^

5

6 ^1^Institute of Avian Research, An der Vogelwarte 21, D-26386 Wilhelmshaven, Germany
7 ^2^Centre d’Etudes Biologiques de Chizé, CNRS-Université de La Rochelle, UMR-7372,
8 Villiers-en-Bois, 79360-France

9

10	* Corresponding author: [frederic.angelier@cebc.cnrs.fr](mailto:frederic.angelier@cebc.cnrs.fr)

# 11 Abstract

12 In the last decade, house sparrow populations have shown a general decline, especially in cities.
13 Avian malaria has been recently suggested as one of the potential causes of this decline, and its
14 detrimental effects could be exacerbated in urban habitats. It was initially thought that avian
15 malaria parasites would not have large negative effects on wild birds because of their long co-
16 evolution with their hosts. However, it is now well-documented that they can have detrimental
17 effects at both the primo- and chronical infection stages. In this study, we examined avian
18 malaria infection and its physiological and morphological consequences in four populations of
19 wild house sparrows (2 urban and 2 rural). We did not find differences in proportions of infected
20 individuals among the populations. However, we observed that the proportion of infected
21 individuals increased during the course of the season, and that juveniles were less infected than
22 adults. We did not detect a strong effect of malaria infection on physiological, morphological
23 and condition indexes. Complex parasite dynamics and the presence of confounding factors
24 could have masked the potential effects of infection. Thus, longitudinal and experimental
25 studies are needed to understand the evolutionary ecology of this very common, but still poorly
26 understood, wild bird parasite.

27

# 28 Keywords

29 Avian	malaria,	corticosterone,	haematocrit,	*Haemoproteus*,	hormonal	stress,	*Passer*
30 *domesticus*, passerine, *Plasmodium*, urbanization.

# 31 Introduction

32 Avian haemosporidian	parasites	consist of three	genera	(*Plasmodium, Haemoproteus,*
33 *Leucocytozoon*) transmitted by different insect vectors to birds [1], and have been extensively
34 studied in the last few decades as a model in evolutionary ecology [2]. Avian haemosporidian
35 parasites can be found in numerous species worldwide and their prevalence can reach very high
36 percentages in some wild bird populations [3–9]. Due to a long co-evolution with their hosts, it
37 was initially suggested that these parasites would not have large negative effects on wild birds
38 [10]. On the other hand, these parasites had a huge detrimental effect on naive bird populations,
39 especially following their introduction (e.g. the introduction of *Plasmodium relictum* and its
40 vector *Culex quinquefasciatus* to the Hawaiian islands [11–13]).
41 The effects of avian malaria vary throughout the course of the infection. Following the primo infection, the host experiences an acute phase with a high level of parasitaemia [1], with
42 important red blood cell destructions and tissue damages caused by the parasite development
43 [14]. Infected individuals suffer from substantial costs, such as reduced activity [15], impaired
44 growth [16], reduced immunity [17], poor body condition [18], anaemia [19,20], and even death
45 [21–23]. If the bird survives this acute phase, the infection enters into a chronic phase,
46 characterized	by	a	much	lower	parasitaemia	alternates	with	cycles	of parasitaemia
47 recrudescence [24,25]. This phase is also associated with significant costs, [20,22], such as
48 reduced breeding effort [26] and performance [27–29].
49 In the last decade, urban house sparrow populations have declined in many cities [30–
50 33], and interestingly, avian malaria parasitaemia has recently been suggested as a possible
51 cause of these population collapses [34]. Across avian species, there is mixed evidence for a
52 difference	in	prevalence	of	haemosporidian	parasites	between	urban	and non-urban
53 populations. While some studies found a higher prevalence in urban habitats [35–37], others
54 found no difference [38] or even a lower prevalence [39–41]. Regarding house sparrows, one
55 study found no difference in prevalence between urban and rural areas [7], while another study
56 found a higher prevalence in non-urban birds, using both field and experimental data [42].
57 Nevertheless, the detrimental	effect	of malaria on survival	and reproduction could be
58 exacerbated in cities because of additional urban environmental constraints. Accordingly,
59 several studies have shown that urbanization is associated with multiple morphological and
60 physiological changes in house sparrows. For example, urban sparrows are usually smaller, in
61 poorer condition [43–45], and have a lower quality plumage [46] than rural ones. They also
62 suffer from higher oxidative stress, and higher stress levels than rural sparrows [47–49]. These
63 changes have been related to low food availability and quality [43,44,50, but see 51], high rates
64 of pollution [47,51], disturbance (traffic noise for example [52]), or even to a recent increase in
65 predator pressure [53,54]. However, the potential impact of malaria infection on morphological
66 and physiological attributes has to our knowledge never been examined in urban house
67 sparrows (but see [20] in red-winged blackbirds, *Agelaius phoeniceus*).
68 In this study, we examined avian malaria infection and its physiological and
69 morphological correlates in four populations of wild house sparrows (2 urban and 2 rural). First,
70 we investigated how malaria infection differs (1a) between urban and rural populations, (1b)
71 throughout the breeding season, and (1c) between juvenile and adult sparrows. Because
72 previous	studies	comparing	blood	parasite	prevalence	between	urban	and non-urban
73 populations produced mixed results, it was difficult to make predictions for the present study.
74 Nevertheless, we expected that malaria infection would increase throughout the breeding
75 season, since ambient temperature seems to be an important prevalence predictor, in temperate
76 areas [55]. Similarly, we predicted that malaria infection would be higher in adults compared
77 to juveniles because adults could have been infected during previous seasons [7]. Second, we
78 explored the potential links between malaria infection status and several morphological (body
79 size, body mass), physiological (stress hormones levels, haematocrit) and condition (fat and
80 muscle scores, body condition) attributes. We tested whether infection status could (2a) affect
81 the physiology, the morphology and the condition of house sparrows and (2b) account for the
82 differences between urban and rural sparrows. In a previous paper using the same data set [44],
83 we reported that urban house sparrows were in poorer body condition than rural sparrows, and
84 a previous study suggested that malaria infection can reduce survival in adult and juvenile house
85 sparrows [34]. Accordingly, we predicted that malaria infection would impair growth (i.e.
86 reduced body size) and would be associated with a poor health status (i.e. low body condition,
87 low haematocrit, high stress hormones levels). Avian malaria infection seems to be particularly
88 detrimental following the primo infection [21,56,57], which is likely to occur at the juvenile
89 stage when sparrows are especially sensitive to environmental and urban-related constraints
90 [58,59]. Therefore, we predicted that malaria infection would be especially detrimental in
91 juveniles compared to adults. Finally, we also predicted that the detrimental influence of avian
92 malaria would be exacerbated for urban sparrows because of the additional and cumulative
93 constraints of the urban environment [43,44,47,53].

95

# 96 Material and methods

97

### 98 Ethics statement

99 This work was conducted according to all institutional and national guidelines for animal care
100 and use. The experimental protocols have been approved by the ethics committee of Poitou-
101 Charentes, France (authorization number: CE2012-7). The permit for capture, sampling and
102 banding was delivered by the ‘Centre de Recherches sur la Biologie des Populations d’Oiseaux’
103 (National Museum of Natural History, Paris) (permit number: 13794). The permits to sample
104 public areas (CEBC and La Rochelle populations) were delivered by the ‘Préfecture de la
105 Charente-Maritime’, the ‘Préfecture des Deux-Sèvres’ and the ‘Centre d’Etudes Biologiques
106 de Chizé’ (hereafter CEBC).

### 108 Study sites and sampling

109 We captured 113 house sparrows (68 adults and 45 juveniles) from four populations located in
110 Western France (Table 1, Fig 1) using mist-nets during the 2013 breeding season (11th of May
111 - 23rd	of August) in the context of a previous study [44]. Two populations	were located in medium-sized cities: La Rochelle (46°08’52.8”N, 1°09’12.7”W, 75,000 inhabitants) and Niort

112	(46°18’46.4”N, 0°28’44.3”W, 58,000 inhabitants) (Fig 1, Table 1). The two other populations

113 were located in rural habitats, either in a village (Villefollet, 46°07’37.7”N, 0°16’04.4”W,
114 200 inhabitants) or at a research station surrounded by a forest (CEBC, 46°08’50.5”N,
115 0°25’34.2”W) (Fig 1, Table 1).
116 Within 3 minutes after capture, we collected a blood sample from the brachial vein of
117 each bird (150µl) to measure ‘baseline corticosterone’ levels [60]. The blood collected will also
118 be used to measure haematocrit and for molecular analyses (see below). We collected a second
119 blood sample (150µl) after 30 minutes, to measure ‘stress-induced corticosterone’ levels [61].
120 Then, we banded birds with a numbered metal band, and measured their weight (hereafter ‘body
121 mass’), wing, and tarsus length (all individuals measured by one experimenter, A.M.). We
122 determined fat and muscle scores as previously described [58,62]. We calculated an index of
123 body condition using the residuals of the linear regression of body mass vs. tarsus length (*F*1,111

124	= 45.98, p < 0.001, *R2* = 0.49). We determined the age of the birds (adult or juvenile) based on

125 plumage characteristics [63], and then released birds at their site of capture. In the case of an
126 individual was recaptured, we immediately released it, to avoid any useless additional stress.

### 128 Physiological measurements

129 To measure haematocrit (determined by one experimenter, A.M.), we centrifuged 20µl of whole
130 blood in a heparinized micro-capillary tube at 11,000 rpm for 3 minutes, and calculated the ratio
131 between the volume of red blood cells and the total volume of whole blood (n = 113).
132 For each bird, we centrifuged blood samples (4,500 rpm, 7 min), separated plasma from
133 red blood cells, transferred samples to separate tubes, and stored them at -20°C until analyses.
134 We measured plasma concentration of corticosterone, from the samples collected 3
135 and 30 minutes after capture, using a radio-immunoassay, in duplicate, according to
136 the protocol developed by [64]. The minimum detectable corticosterone level was 0.28 ng.ml-
137 ^1^, and the intra- and inter-assay coefficients of variation were 8.27% and 12.01%, respectively.

### 139 Molecular analyses

140 We also stored red blood cells (approx. 75 µl) at -20°C until analyses. We extracted DNA from
141 red blood cells using the commercial kit NucleoSpin Blood (Macherey-Nagel Gmbh, Germany)
142 and followed the provided instructions (n = 113). We used a Nanodrop (ND-1000) to
143 standardize the concentration of the extracted DNA at 20ng.µl^-1^, and used these standardized
144 samples for molecular sexing and parasite screening analyses.

*Molecular sexing*

145 We determined the sex of adults visually (n = 68) [63], but determined the sex of juveniles (n

147	= 45) using the molecular technique described in [65].

149 *Parasite screening*
150 We detected the presence of blood parasites using a nested PCR [66], which targets the
151 cytochrome *b* gene of *Plasmodium*, *Leucocytozoon* and *Haemoproteus* in the extracted DNA
152 (n = 113). We slightly modified the protocol described in [66] by using 40ng of total genomic
153 DNA at 20ng. µl^-1^, 0.8mM of each dNTP, 3.5mM of MgCl2, 1.2µM of each primer and 0.625
154 units of Taq polymerase G2 Hot Start (Promega). The first PCR amplified a 580-bp-long
155 fragment from the three blood parasite genera (primers HaemNFI/HaemNR3 [67,68]). For the
156 second	PCRs, which	amplified	a	524-bp-long	fragment, a	first	couple	of	primers
157 (HaemF/HaemR2 [67,68]) was used to detect indifferently *Plasmodium* or *Haemoproteus*, and
158 a second couple of primers (HaemFL/HaemR2L [66]) to detect *Leucocytozoon*. This method
159 is highly repeatable, with a minimum limit of detection of one infected blood cell per 100,000.
160 We ran the products of the amplifications on a 1.8% agarose gel at 100V for 1h30, and
161 visualized them with an ethidium bromide stain under ultraviolet light. We tested negative
162 samples twice to minimize false negatives. We did not detect any positive blood sample for
163 *Leucocytozoon*.

### 165 Statistical analyses

166 To investigate which factors could predict blood parasite infection, we used a Generalized
167 Linear Model (GLM) with a logit link function and a variance given by a binomial distribution.
168 The infection status of the individual (infected or uninfected) was the dependent variable and
169 the age (categorical, adult or juvenile), the sex (categorical, male or female) of the bird, and the population (categorical, four levels), were the explanatory variables. We also added
170 capture date (standardized Julian date) as a covariate. To test if the effects of age and sex were consistent across the breeding season and the different populations, we also considered
171 all first-order interactions.
172 To test if blood parasite infection could predict physiological, morphological and
173 condition measures, we built nine different Linear Models (LMs) using one of the physiological
174 parameters (i.e. haematocrit, baseline corticosterone levels, and stress-induced corticosterone
175 levels), morphological measures (i.e. body mass, wing, tarsus), or condition indexes (i.e. fat
176 score, muscle score, and body condition) as the dependent variable. The models with fat and
177 muscle scores as dependent variables were, however, two GLMs fitted with a Poisson
178 distribution. Infection status (infected or uninfected) was added as the explanatory variable and
179 population, age, and sex were added as covariates. To test if the effect of the infection status
180 was consistent across ages, sexes, populations and breeding season, we also considered all first-
181 order interactions, as well as the second-order interaction between infection status, age, and
182 sex.
183 In our dataset, there was collinearity between population, age, and capture date
184 (Variance inflation factors > 1/(1-*R^2^*) [69]) due to the absence of juveniles and the absence of
185 sampling in the La Rochelle population at the beginning of the season (from mid-May to mid-
186 June). To limit this problem, we also ran LMs similar to those presented above, but separating
187 adults (n = 68) and juveniles (n = 45) (See Supplemental Information). One of the sampled
188 juveniles was an outlier with a small body mass and short wing length. We ran our models with
189 and without this individual and it did not modify our conclusions.
190 All models were run with R 3.6.0 [70] using the functions ‘glm and ‘lm’ implemented
191 in the package ‘lme4’ [71] using restricted maximum likelihood estimates of the parameters,
192 and by conducting a type III Analysis of Variance (ANOVA) for LMs and likelihood ratio (LR)
193 tests for GLMs using the package ‘car’ [72]. Non-significant interactions were removed from
194 the presented models in the Results, with the level of significance set to α = 0.05, to allow for
195 a straightforward interpretation of the effect of single terms. Parameter estimates are given as
196 the mean ± 1 standard error (SE). The coefficient of determination were calculated for all the
197 models using the function ‘r.squaredGLMM’ implemented in the package ‘MuMIn’ [73].

# 199 Results

200 *Blood parasite infection in house sparrows*
201 The number of infected individuals did not differ among populations (LR Chi2 = 0.42, df = 3,
202 p = 0.935; Fig 2a) nor between urban and rural populations (LR Chi^2^ = 0.04, df = 1, p = 0.835).
203 The number of infected individuals increased during the breeding season, from May to August
204 (Table 2, Fig 2b). Juvenile birds were less infected than adults (Table 2, Fig 2).
205 In juveniles, the number of infected individuals increased during the breeding season
206 (Table S1; Fig. 2b), and male juveniles were more infected than female juveniles (Table S1).
207 In adults, the number of infected individuals marginally increased during the breeding
208 season (β = 0.50 ± 0.27, Z-value = 1.85, p = 0.064; Table S1; Fig. 2b) but did not differ between
209 sexes (Table S1).
211 *Relationships between parasite infection and physiology*
212 Infection status was not associated with any of the three physiological parameters investigated
213 (haematocrit, baseline corticosterone levels, and stress-induced corticosterone levels, Table 3a,
214 Figs 3a, b, c).
215 We also did not find an association between infection status and physiology in the
216 models where adults and juveniles were analysed separately (Table S2a). We could mentioned,
217 however, the marginal relationship between infection status and haematocrit in juveniles
218 (infected juveniles had a slightly lower haematocrit than uninfected ones, β = -0.03 ± 0.02, t-

220	value = -1.85, p = 0.072, Table S2a; Fig. 3a).

221

222 *Relationships between parasite infection and morphology*
223 Infection status did not predict any of the three morphological measures (body mass, tarsus
224 length, wing length, Table 3b, Figs 3d, e, f). We detected an interaction between infection status,
225 age and sex for the body mass (*F*1,100 = 4.63, p = 0.034, Table 3b) and for the tarsus length (*F*1,98

226	= 4.54, p = 0.036, Table 3b), indicating that the slopes of body mass and tarsus length between

227 infected and uninfected individuals varied between ages and sexes (Figs 3d, e).
228 When adults were analysed separately, the infection status was not associated with body
229 mass, tarsus length, nor wing length (Table S2b). We observed an interaction between infection
230 status and sex for the body mass (*F*1,60 = 4.87, p = 0.031, Table S2b, Fig S1a) and for the tarsus
231 length (*F*1,60 = 4.38, p = 0.041, Table S2b, Fig S1b). However, post-hoc pairwise comparisons
232 indicated that body mass and tarsus length did not differ between infected and uninfected
233 females (mean ± SE of body mass for infected females = 26.2 ± 0.48, mean ± SE of body mass
234 for uninfected females = 27.3 ± 0.45, t-ratio = 1.10, p = 0.693; mean of tarsus length for infected
235 females = 18.3 ± 0.19, mean of tarsus length for uninfected females = 19.0 ± 0.22, t-ratio =
236 1.66, p = 0.352), or between infected and uninfected males (mean of body mass for infected
237 males = 27.7 ± 0.48, mean of body mass for uninfected males = 26.8 ± 0.30, t-ratio = -2.03, p

238	= 0.190; mean of tarsus length for infected males = 19.0 ± 0.23, mean of tarsus length for

239	uninfected males = 18.7 ± 0.25, t-ratio = -1.27, p = 0.585).

240 When juveniles were analysed separately, we found that infected juveniles had a longer
241 tarsus than uninfected individuals (Table S2b, mean ± SE for infected juveniles = 18.5 ± 0.16,
242 mean ± SE for uninfected juveniles = 17.9 ± 0.11; Fig 3e). In juveniles, we also found an
243 interaction between infection status and capture date for tarsus length (F1,37 = 6.86, p = 0.013,
244 Table S2b), probably because both juvenile size and malaria infection increased throughout the
245 breeding season (Fig 2b).
247 *Relationships between parasite infection and condition*
248 Infection status was not associated with sparrow condition (body condition, fat, and muscle
249 scores, Table 3c). We only found an interaction between infection status and age for the body
250 condition index (*F*1,104 = 4.24, p = 0.042, Table 3c), indicating that the slope of body condition
251 between infected and uninfected individuals varied between adults and juveniles (Fig 3g).
252 When adults were analysed separately, infection status was not associated with body
253 condition (Table S2c). We observed an interaction between infection status and population for
254 the body condition index (*F*3,58 = 2.91, p = 0.042, Table S2c). For all the populations, infected
255 adults had a lower body condition than uninfected adults, except in La Rochelle (Fig S2).
256 When juveniles were analysed separately, we found an association between infection
257 status and body condition (Table S2c), indicating that infected juveniles had a higher body
258 condition than uninfected juveniles (mean ± SE of body condition for infected juveniles = 0.24
259 ± 0.12, mean of body condition ± SE for uninfected juveniles = -0.12 ± 0.09; Fig 3g).

# 261 Discussion

262 *Blood parasite infection in house sparrows*
263 Our study, conducted in urban and rural wild populations of house sparrows, provides several
264 pieces of information regarding blood parasite infection in this species. The number of infected
265 individuals did not differ among the four populations, regardless of whether populations were
266 urban or rural. Similarly, other previous studies did not find differences in prevalence between
267 urban and rural habitats [38], even in the same biological model [7]. However, other contrasting
268 results prevent us from suggesting a conclusive pattern [35–37,39–42]. Changes in climatic
269 conditions (mainly temperature and precipitation) and habitat characteristics can affect both
270 vector-borne parasites and their insect vectors (e.g. reproductive cycle), and thus affect
271 prevalence [36,55,74–76]. However, the populations investigated in this study are probably too
272 close geographically to represent marked differences in climate (but see for instance [77]). One
273 might also argue that the sampled cities are not large enough to observe any effect of
274 urbanization. Yet, previous studies reported morphological and physiological differences
275 between the same urban and rural populations [44,49]. Accordingly, at a larger scale of
276 sampling, the degree of urbanization did not correlate with *Plasmodium* prevalence in house
277 sparrows either, even if larger cities were included [7]. Altogether, these studies seem to
278 indicate that malaria prevalence would not be strongly affected by urbanization in house
279 sparrows.
280 If prevalence can be affected by climatic conditions, it is expected that seasonality
281 would explain a significant amount of the variation in malaria prevalence, especially in
282 temperate areas, where vector abundance and susceptible host availability are likely to vary
283 [78,79]. Indeed, we observed that the number of infected individuals increased during the
284 breeding season (May-August). This could be explained by an increase in vector abundance in
285 the summer, as well as an increase in the number of fledglings and juveniles that are susceptible
286 to being primo-infected. This latter hypothesis seems to be confirmed by an overall increase in
287 the number of infected individuals during the course of the breeding season and by a higher
288 number of infected individuals in juveniles than in adults, which has also been observed in
289 previous studies [7,80].
291 *Relationships between parasite infection and physiology*
292 We	did	not	detect	any	physiological	(i.e. haematocrit, baseline	and	stress-induced
293 corticosterone) differences between infected and uninfected birds. Some studies, also in natural
294 population, have investigated the association between physiological variables and	blood
295 parasite infection, and they produced mixed results [20,79,81–86].
296 Several previous studies observed a lower haematocrit in infected birds compared to
297 uninfected birds [17,20,81,82,87]. Haematocrit is usually considered as a relevant marker
298 integrating both red cell damage due to parasites, as well as costs of the immune response
299 [87,88], including destruction of like red blood cells by T-cell activity [89,90]. In agreement,
300 our analyses detected a trend for infected juveniles to have a lower haematocrit than uninfected
301 juveniles, but the absence of significance prevents any conclusion. It is possible that heavily
302 infected juveniles with a low haematocrit die quickly and thus not sampled.
303 In previous studies, experimental elevation of corticosterone reduced parasite resistance
304 (e.g. for	avian	malaria: [91,92]), possibly	due	to	the	immunosuppressive	effect of
305 glucocorticoids [93]. However, this result has not always been confirmed in observational
306 studies [79,82,83]. Other studies	also found that higher	corticosterone levels increased
307 haemosporidian parasite tolerance [82], possibly due to tissue repair enhancement and/or
308 damage limitations [93–95]. In addition, malaria could also lead to energetic costs, which may
309 translate into increased corticosterone levels [96–98]. Here, we did not find any relationship
310 between malaria infection and baseline or stress-induced corticosterone levels, suggesting that
311 Hypothalamic-pituitary-adrenal axis function is not dramatically affected by malaria infection.
312 In line with the present study, previous investigations often failed to detect any associations
313 between infection and corticosterone levels [20,82,99], while significant, but mixed results
314 were found when investigating the relationship between corticosterone levels and parasite

315	intensity [79,82,83].

316

317 *Relationships between parasite infection, condition, and morphology*
318 According to the significant interactions that we detected, the relationships between blood
319 parasite infection and	morphology and	condition appear	to be complex. Intricate and
320 inconsistent	findings	were	previously	reported	in	several	studies
321 [6,7,14,17,20,81,86,87,100,101]. This may be because hosts in good condition may be able to
322 allocate enough energy to trigger efficient immune responses; however, good condition may
323 provide more resources for the parasite, which could increase virulence without leading to
324 mortality [102–104]. Overall, our results indicate that infected juveniles are larger and in better
325 condition than uninfected juveniles, and a similar result was found in another study on the same
326 species [45]. Blood parasites may be associated with an increased risk of mortality [21,23,80],
327 especially during primo infection, and this risk may increase when juveniles are in poor
328 condition or not fully grown (small and light individuals, [16,18,105]). This may explain why
329 we, unexpectedly, found that infected juveniles were larger and in better condition that
330 uninfected ones. Conversely, in adults, infected birds were overall in poorer condition than
331 uninfected individuals, suggesting that infection may have energetic costs (immune response,
332 lower activity and feeding, and higher metabolism [17,22,92]).
334 *Underlying hypotheses*
335 The absence of strong detectable effects of parasite infections on house sparrow physiology,
336 morphology, and condition could be explained by three main hypotheses. First, the seasonality
337 of blood parasite emergence could create co-variation with some factors, such as bird age, chick
338 emancipation and sampling date, which could decrease statistical power and mask existing
339 effects. Second, the sampled populations could be highly tolerant to the parasite, which prevent
340 the detection of any effects, although the regional cause of such potential tolerance remains
341 difficult to explain. Tolerance could be an adaptive strategy to minimize the negative impact of
342 infection and the cost of resistance, and should be particularly suitable for endemic and chronic
343 diseases [106,107], such as haemosporidian parasites in the studied area [1,108]. However, the
344 balance between tolerance and resistance is still poorly understood for these parasites
345 [82,86,92]. Third, strong selection pressures imposed by blood parasites could also explain the
346 absence of results. In field studies, only birds that are able to fly can be caught and sampled.
347 Infected birds that exhibit substantial disease symptoms may be completely absent from the
348 study due to considerably reduced their activity levels [15] or because they died quickly after
349 infection [21]. The remaining infected birds sampled could be the highest quality individuals,
350 in which physiological costs of infection are below the detection threshold. In support with this
351 hypothesis, one of the few significant results that we found was that infected juveniles were in
352 better condition than uninfected juveniles, such as in [45]. This makes sense because juveniles
353 are more likely to be primo-infected, and infected at higher intensities, than adults [1].
354 Furthermore, studies conducted in the UK observed that house sparrow declines are associated
355 with reduced juvenile recruitment linked to lower overwinter survival [109,110] and that
356 the intensity of blood parasite infection reduced survival, even if this effect was also
357 detected in adults [34].
358 Therefore, it would also be interesting to measure parasite intensities in our house sparrow
359 samples to test how the parameters measured are correlated with parasitaemia and would
360 corroborate the study conducted in the UK [34].

# 361 Conclusion

362 To conclude, this study highlights the complexity of avian haemosporidan parasite dynamic as
363 well as the difficulty to detect potential associated costs, especially in natural populations.
364 Seasonality, selective disappearance of infected individuals of different quality, age or sex, and
365 habitat-associated factors could interact and reduce our capacity to detect the effects of blood
366 parasites, if there are any. Thus, the statistical power, limited in our study, has to be large
367 enough to be able to test (and detect) these multiple interactions. Moreover, avian
368 haemosporidian parasites consist of three genera comprising many species and strains
369 [68,111,112], which could be different in term of biology, ecology and virulence [1,113]. Co-
370 infections between two or more blood parasite lineages could also have amplified negative
371 effects	[114,115]. Considering	these	different	levels	of	diversity would	improve our
372 understanding about these parasites and their effects on wild populations. In natural
373 populations, longitudinal sampling during the course of the year (e.g. including the non-
374 breeding season [81]) could provide valuable information on parasite dynamics and associated
375 selective processes. A longitudinal survey could also be carried out using experimental
376 approaches that simulate different environments (e.g. urban versus rural) and could provide
377 knowledge about the evolutionary ecology of this very common, but still poorly understood,
378 wild bird parasite.

# 380 Acknowledgements

381 We warmly thank Dr G. Sorci for providing us positive house sparrow samples for *Plasmodium*.
382 We thank E. Beaugeard for her help with DNA extraction and Dr S. Hope for carefully editing
383 this manuscript.

# 385 Authors’ contributions

386 F.A., F.B. and C.B. designed the study. F.A., F.B., A.M. collected the data. C.R., C.P., and C.B.
387 conducted the laboratory analyses. C.B. performed the statistical analyses. C.B. and F.A. co-
388 wrote the manuscript with contributions from all co-authors.

# 390 References

391 1.	Valkiūnas G. Avian malaria parasites and other haemosporidia. Valkiūnas, Gediminas;
392 Institute of Ecology, Vilnius University, Lithuania: CRC Press; 2005.
393 2.	Rivero A, Gandon S. Evolutionary Ecology of Avian Malaria: Past to Present. Trends

394	Parasitol. 2018;34: 712–726. doi:10.1016/j.pt.2018.06.002

395 3.	Mendes L, Piersma T, Lecoq M, Spaans B, Ricklefs RE. Disease-limited distributions?
396 Contrasts in the prevalence of avian malaria in shorebird species using marine and

397	freshwater habitats. Oikos. 2005;109: 396–404. doi: [10.1111/j.0030-1299.2005.13509.x](https://doi.org/10.1111/j.0030-1299.2005.13509.x)

398 4.	Ribeiro SF, Sebaio F, Branquinho FCS, Marini MA, Vago AR, Braga EM. Avian malaria
399 in Brazilian passerine birds: parasitism detected by nested PCR using DNA from stained

400	blood smears. Parasitology. 2005;130: 261–267. doi:10.1017/S0031182004006596

401	5.	Loiseau C, Harrigan RJ, Robert A, Bowie RCK, Thomassen HA, Smith TB, et al. Host

402	and habitat specialization of avian malaria in Africa. Mol Ecol. 2012;21: 431–441.

403	doi:10.1111/j.1365-294X.2011.05341.x

404	6.	Bichet C, Scheifler R, Coeurdassier M, Julliard R, Sorci G, Loiseau C. Urbanization,

405	Trace Metal Pollution, and Malaria Prevalence in the House Sparrow. PLoS One. 2013;8:

406	e53866. doi:10.1371/journal.pone.0053866

407	7.	Bichet C, Sorci G, Robert A, Julliard R, Lendvai AZ, Chastel O, et al. Epidemiology of

408	*Plasmodium Relictum* Infection in the House Sparrow. J Parasitol. 2014;100: 59–65.

409	doi:10.1645/12-24.1

410	8.	Gonzalez AD, Matta NE, Ellis VA, Miller ET, Ricklefs RE, Rafael Gutierrez H. Mixed

411	Species Flock, Nest Height, and Elevation Partially Explain Avian Haemoparasite

412	Prevalence	in	Colombia.	PLoS	One.	2014;9:	e100695.

413	doi:10.1371/journal.pone.0100695

414	9.	Lutz HL, Hochachka WM, Engel JI, Bell JA, Tkach VV, Bates JM, et al. Parasite

415	Prevalence Corresponds to Host Life History in a Diverse Assemblage of Afrotropical

416	Birds	and	Haemosporidian	Parasites.	PLoS	One.	2015;10:	e0121254.

417	doi:10.1371/journal.pone.0121254

418 10.	Fallis AM, Desser SS. On species of Leucocytozoon, Haemoproteus, and Hepatocystis.
419 Keier, J.P. Parasitic Protozoa. Keier, J.P. New York; 1977. pp. 239–266.
420 11.	Scott JM, Mountainspring S, Ramsey FL, Kepler CB. Forest Bird Communities of the
421 Hawaiian Islands: Their Dynamics, Ecology, and Conservation. Studies in Avian
422 Biology. 1986. Available[: http://pubs.er.usgs.gov/publication/5200067](http://pubs.er.usgs.gov/publication/5200067)
423 12.	Atkinson	CT, Woods KL, Dusek RJ, Sileo LS, Iko WM. Wildlife disease	and
424 conservation in Hawaii: Pathogenicity of avian malaria (*Plasmodium relictum*) in
425 experimentally infected Iiwi (*Vestiaria coccinea*). Parasitology. 1995;111: S59–S69. doi:

426	[10.1017/s003118200007582x](https://doi.org/10.1017/s003118200007582x)

427	13.	Samuel MD, Hobbelen PHF, DeCastro F, Ahumada JA, Lapointe DA, Atkinson CT, et

428	al. The dynamics, transmission, and population impacts of avian malaria in native

429	Hawaiian birds: a modeling approach. Ecol Appl. 2011;21: 2960–2973. doi: [10.1890/10-](https://doi.org/10.1890/10-1311.1)

430	[1311.1](https://doi.org/10.1890/10-1311.1)

431	14.	Atkinson CT, Dusek RJ, Woods KL, Iko WM. Pathogenicity of avian malaria in

432	experimentally-infected	Hawaii	Amakihi.	J	Wildl	Dis.	2000;36:	197–204.

433	doi:10.7589/0090-3558-36.2.197

434	15.	Yorinks N, Atkinson CT. Effects of malaria on activity budgets of experimentally

435	infected juvenile Apapane (*Himatione sanguinea*). The Auk. 2000;117: 731–738. doi:

436	[10.1093/auk/117.3.731](https://doi.org/10.1093/auk/117.3.731)

437	16.	Calero-Riestra M, Garcia JT. Sex-dependent differences in avian malaria prevalence and

438	consequences of infections on nestling growth and adult condition in the Tawny pipit,

439	*Anthus campestris*. Malar J. 2016;15: 178. doi:10.1186/s12936-016-1220-y

440	17.	Ellis VA, Cornet S, Merrill L, Kunkel MR, Tsunekage T, Ricklefs RE. Host immune

441	responses to experimental infection of Plasmodium relictum (lineage SGS1) in domestic

442	canaries (*Serinus canaria*). Parasitol Res. 2015;114: 3627–3636. doi:10.1007/s00436-

443	015-4588-7

444	18.	Marzal A, Asghar M, Rodriguez L, Reviriego M, Hermosell IG, Balbontin J, et al. Co-

445	infections by malaria parasites decrease feather growth but not feather quality in house

446	martin. J Avian Biol. 2013;44: 437–444. doi:10.1111/j.1600-048X.2013.00178.x

447	19.	Palinauskas V, Valkiūnas GN, Bolshakov CV, Bensch S. Plasmodium relictum (lineage

448	P-SGS1): Effects on experimentally infected passerine birds. Exp Parasitol. 2008;120:

449	372–380. doi:10.1016/j.exppara.2008.09.001

450	20.	Schoenle LA, Kernbach M, Haussmann MF, Bonier F, Moore IT. An experimental test

451	of the physiological consequences of avian malaria infection. J Anim Ecol. 2017;86:

452	1483–1496. doi:10.1111/1365-2656.12753

453	21.	Krams IA, Suraka V, Rantala MJ, Sepp T, Mierauskas P, Vrublevska J, et al. Acute

454	infection of avian malaria impairs concentration of haemoglobin and survival in juvenile

455	altricial birds. J Zool. 2013;291: 34–41. doi:10.1111/jzo.12043

456	22.	Asghar M, Hasselquist D, Hansson B, Zehtindjiev P, Westerdahl H, Bensch S. Hidden

457	costs of infection: Chronic malaria accelerates telomere degradation and senescence in

458	wild birds. Science. 2015;347: 436–438. doi:10.1126/science.1261121

459	23.	Townsend AK, Wheeler SS, Freund D, Sehgal RNM, Boyce WM. Links between blood

460	parasites, blood chemistry, and the survival of nestling American crows. Ecol Evol.

461	2018;8: 8779–8790. doi:10.1002/ece3.4287

462	24.	Bensch S, Waldenstrom J, Jonzen N, Westerdahl H, Hansson B, Sejberg D, et al.

463	Temporal dynamics and diversity of avian malaria parasites in a single host species. J

464	Anim Ecol. 2007;76: 112–122. doi:10.1111/j.1365-2656.2006.01176.x

465	25.	Asghar M, Westerdahl H, Zehtindjiev P, Ilieva M, Hasselquist D, Bensch S. Primary

466	peak and chronic malaria infection levels are correlated in experimentally infected great

467	reed warblers. Parasitology. 2012;139: 1246–1252. doi:10.1017/S0031182012000510

468	26.	Marzal A, de Lope F, Navarro C, Møller AP. Malarial parasites decrease reproductive

469	success: an experimental study in a passerine bird. Oecologia. 2005;142: 541–545.

470	doi:10.1007/s00442-004-1757-2

471	27.	Marzal A, Bensch S, Reviriego M, Balbontin J, de Lope F. Effects of malaria double

472	infection in birds: one plus one is not two. J Evol Biol. 2008;21: 979–987.

473	doi:10.1111/j.1420-9101.2008.01545.x

474	28.	Asghar M, Hasselquist D, Bensch S. Are chronic avian haemosporidian infections costly

475	in wild birds? J Avian Biol. 2011;42: 530–537. doi:10.1111/j.1600-048X.2011.05281.x

476	29.	Romano A, Nodari R, Bandi C, Caprioli M, Costanzo A, Ambrosini R, et al.

477	Haemosporidian parasites depress breeding success and plumage coloration in female

478	barn	swallows	*Hirundo	rustica*.	J	Avian	Biol.	2019;50:	UNSP	e01889.

479	doi:10.1111/jav.01889

480	30.	Summers-Smith JD. The decline of the House Sparrow: A review. British Birds.

481	2003;96: 439–446.

482	31.	Robinson RA, Siriwardena GM, Crick HQP. Size and trends of the House Sparrow

483	*Passer	domesticus*	population	in	Great	Britain.	Ibis.	2005;147:	552–562.

484	doi:10.1111/j.1474-919x.2005.00427.x

485	32.	De Laet J, Summers-Smith JD. The status of the urban house sparrow *Passer domesticus*

486	in	north-western	Europe:	a	review.	J	Ornithol.	2007;148:	S275–S278.

487	doi:10.1007/s10336-007-0154-0

488	33.	De Coster G, De Laet J, Vangestel C, Adriaensen F, Lens L. Citizen science in action-

489	Evidence for long-term, region-wide House Sparrow declines in Flanders, Belgium.

490	Landsc Urban Plan. 2015;134: 139–146. doi:10.1016/j.landurbplan.2014.10.020

491	34.	Dadam D, Robinson RA, Clements A, Peach WJ, Bennett M, Rowcliffe JM, et al. Avian

492	malaria-mediated population decline of a widespread iconic bird species. R Soc open sci.

493	2019;6: 182197. doi:10.1098/rsos.182197

494	35.	Belo NO, Pinheiro RT, Reis ES, Ricklefs RE, Braga EM. Prevalence and Lineage

495	Diversity of Avian Haemosporidians from Three Distinct Cerrado Habitats in Brazil.

496	PLoS One. 2011;6: e17654. doi:10.1371/journal.pone.0017654

497	36.	Hernández-Lara C, Gonzalez-Garcia F, Santiago-Alarcón D. Spatial and seasonal

498	variation of avian malaria infections in five different land use types within a Neotropical

499	montane	forest	matrix.	Landsc	Urban	Plan.	2017;157:	151–160.

500	doi:10.1016/j.landurbplan.2016.05.025

501	37.	Horak P, Ots I, Vellau H, Spottiswoode C, Moller AP. Carotenoid-based plumage

502	coloration reflects hemoparasite infection and local survival in breeding great tits.

503	Oecologia. 2001;126: 166–173. doi:10.1007/s004420000513

504	38.	Fokidis HB, Greiner EC, Deviche P. Interspecific variation in avian blood parasites and

505	haematology associated with urbanization in a desert habitat. J Avian Biol. 2008;39:

506	300–310. doi:10.1111/j.2008.0908-8857.04248.x

507	39.	Bentz S, Rigaud T, Barroca M, Martin-Laurent F, Bru D, Moreau J, et al. Sensitive

508	measure of prevalence and parasitaemia of haemosporidia from European blackbird

509	(*Turdus merula*) populations: value of PCR-RFLP and quantitative PCR. Parasitology.

510	2006;133: 685–692. doi:10.1017/S0031182006001090

511	40.	Geue D, Partecke J. Reduced parasite infestation in urban Eurasian blackbirds (Turdus

512	merula):	a	factor	favoring	urbanization?	Can	J	Zool.	2008;86:	1419–1425.

513	doi:10.1139/Z08-129

514	41.	Okanga S, Cumming GS, Hockey PAR, Peters JL. Landscape structure influences avian

515	malaria ecology in the Western Cape, South Africa. Landsc Ecol. 2013;28: 2019–2028.

516	doi:10.1007/s10980-013-9949-y

517	42.	Santiago-Alarcón D, Carbó‐ Ramírez P, Macgregor‐Fors I, Chávez‐Zichinelli CA,

518	Yeh PJ. The prevalence of avian haemosporidian parasites in an invasive bird is lower in

519	urban than in non‐ urban environments. Ibis. 2018;162: 201-214. doi:10.1111/ibi.12699

520	43.	Liker A, Papp Z, Bokony V, Lendvai AZ. Lean birds in the city: body size and condition

521	of house sparrows along the urbanization gradient. J Anim Ecol. 2008;77: 789–795.

522	doi:10.1111/j.1365-2656.2008.01402.x

523	44.	Meillere A, Brischoux F, Parenteau C, Angelier F. Influence of Urbanization on Body

524	Size, Condition, and Physiology in an Urban Exploiter: A Multi-Component Approach.

525	PLoS One. 2015;10: e0135685. doi:10.1371/journal.pone.0135685

526	45.	Jiménez-Peñuela J, Ferraguti M, Martínez-de la Puente J, Soriguer R, Figuerola J.

527	Urbanization and blood parasite infections affect the body condition of wild birds. Sci

528	Total Environ. 2019;651: 3015–3022. doi:10.1016/j.scitotenv.2018.10.203

529	46.	Biard C, Brischoux F, Meillere A, Michaud B, Niviere M, Ruault S, et al. Growing in

530	Cities: An Urban Penalty for Wild Birds? A Study of Phenotypic Differences between

531	Urban and Rural Great Tit Chicks (*Parus major*). Front Ecol Evol. 2017;5: UNSP 79.

532	doi:10.3389/fevo.2017.00079

533	47.	Herrera-Dueñas A, Pineda-Pampliega J, Antonio-García MT, Aguirre JI. The Influence

534	of Urban Environments on Oxidative Stress Balance: A Case Study on the	House

535	Sparrow	in	the	Iberian	Peninsula.	Front	Ecol	Evol.	2017;5:	UNSP	106.

536	doi:10.3389/fevo.2017.00106

537	48.	Hudin NS, Teyssier A, Aerts J, Fairhurst GD, Strubbe D, White J, et al. Do wild-caught

538	urban house sparrows show desensitized stress responses to a novel stressor? Biol Open.

539	2018;7: UNSP bio031849. doi:10.1242/bio.031849

540	49.	Beaugeard E, Brischoux F, Henry P-Y, Parenteau C, Trouve C, Angelier F. Does

541	urbanization cause stress in wild birds during development? Insights from feather

542	corticosterone levels in juvenile house sparrows (*Passer domesticus*). Ecol Evol. 2019;9:

543	640–652. doi:10.1002/ece3.4788

544	50.	Gadau A, Crawford MS, Mayek R, Giraudeau M, McGraw KJ, Whisner CM, et al. A

545	comparison of the nutritional physiology and gut microbiome of urban and rural house

546	sparrows (*Passer domesticus*). Comp Biochem Physiol B-Biochem Mol Biol. 2019;237:

547	110332. doi:10.1016/j.cbpb.2019.110332

548	51.	Cid FD, Fernández NC, Perez-Chaca MV, Pardo R, Caviedes-Vidal E, Chediack JG.

549	House sparrow biomarkers as lead pollution bioindicators. Evaluation of dose and

550	exposition length on hematological and oxidative stress parameters. Ecotox Environ

551	Safe. 2018;154: 154–161. doi:10.1016/j.ecoenv.2018.02.040

552	52.	Meillere A, Brischoux F, Ribout C, Angelier F. Traffic noise exposure affects telomere

553	length	in	nestling	house	sparrows.	Biol	Lett.	2015;11:	20150559.

554	doi:10.1098/rsbl.2015.0559

555	53.	Bell CP, Baker SW, Parkes NG, Brooke MDL, Chamberlain DE. The Role of the

556	Eurasian Sparrowhawk (*Accipiter nisus*) in the Decline of the House Sparrow (*Passer*

557	*domesticus*) in Britain. AUK. 2010;127: 411–420. doi:10.1525/auk.2009.09108

558	54.	Thomas RL, Fellowes MDE, Baker PJ. Spatio-Temporal Variation in Predation by Urban

559	Domestic Cats (*Felis catus*) and the Acceptability of Possible Management Actions in

560	the UK. PLoS One. 2012;7: e49369. doi:10.1371/journal.pone.0049369

561	55.	Loiseau C, Harrigan RJ, Bichet C, Julliard R, Garnier S, Lendvai AZ, et al. Predictions

562	of avian Plasmodium expansion under climate change. Sci Rep. 2013;3: 1126.

563	doi:10.1038/srep01126

564	56.	Merino S, Moreno J, Sanz JJ, Arriero E. Are avian blood parasites pathogenic in the

565	wild? A medication experiment in blue tits (*Parus caeruleus*). Proc R Soc B-Biol Sci.

566	2000;267: 2507–2510. doi:10.1098/rspb.2000.1312

567	57.	Knutie SA, Waite JL, Clayton DH. Does avian malaria reduce fledging success: an

568	experimental	test	of	the	selection	hypothesis. Evol	Ecol. 2013;27: 185–191.

569	doi:10.1007/s10682-012-9578-y

570	58.	Leloutre C, Gouzerh A, Angelier F. Hard to fly the nest: A study of body condition and

571	plumage	quality	in	house	sparrow	fledglings. Curr	Zool. 2014;60: 449–459.

572	doi:10.1093/czoolo/60.4.449

573	59.	Meillere A, Brischoux F, Henry P-Y, Michaud B, Garcin R, Angelier F. Growing in a

574	city: Consequences on body size and plumage quality in an urban dweller, the house

575	sparrow	(*Passer	domesticus*).	Landsc	Urban	Plan.	2017;160:	127–138.

576	doi:10.1016/j.landurbplan.2016.12.014

577	60.	Angelier F, Tonra CM, Holberton RL, Marra PP. How to capture wild passerine species

578	to	study	baseline	corticosterone	levels.	J	Ornithol.	2010;151:	415–422.

579	doi:10.1007/s10336-009-0471-6

580	61.	Wingfield J, Deviche P, Sharbaugh S, Astheimer L, Holberton R, Suydam R, et al.

581	Seasonal-Changes of the Adrenocortical Responses to Stress in Redpolls, *Acanthis*

582	*flammea*, in Alaska. J Exp Zool. 1994;270: 372–380. doi:10.1002/jez.1402700406

583	62.	Brown ME. Assessing Body Condition in Birds. Current Ornithology. 1996; 67–135.

584	doi:10.1007/978-1-4615-5881-1_3

585	63.	Anderson TR. Biology of the Ubiquitous House Sparrow: From Genes to Populations.

586	Oxford University Press; 2006.

587	64.	Lormee H, Jouventin P, Trouve C, Chastel O. Sex-specific patterns in baseline

588	corticosterone and body condition changes in breeding Red-footed Boobies *Sula sula*.

589	Ibis. 2003;145: 212–219. doi:10.1046/j.1474-919X.2003.00106.x

590	65.	Fridolfsson A-K, Ellegren H. A Simple and Universal Method for Molecular Sexing of

591	Non-Ratite Birds. Journal of Avian Biology. 1999;30: 116–121. doi:10.2307/3677252

Hellgren O, Waldenstrom J, Bensch S. A new PCR assay for simultaneous studies of Leucocytozoon, Plasmodium, and Haemoproteus from avian blood. J Parasitol. 2004;90: 797–802. doi:10.1645/GE-184R1

| 592 | 66. |
| --- | --- |
| 593 |  |
| 594 |  |
| 595 | 67. |
| 596 |  |
| 597 |  |
| 598 | 68. |
| 599 |  |
| 600 |  |
| 601 | 69. |
| 602 |  |
| 603 | 70. |
| 604 |  |
| 605 |  |
| 606 | 71. |
| 607 |  |
| 608 | 72. |
| 609 | 73. |
| 610 |  |
| 611 | 74. |
| 612 |  |
| 613 |  |
| 614 | 75. |
| 615 |  |
| 616 |  |
| 617 |  |
| 618 | 76. |
| 619 |  |
| 620 | 77. |
| 621 |  |
| 622 |  |
| 623 |  |
| 624 | 78. |
| 625 |  |
| 626 |  |
| 627 | 79. |
| 628 |  |
| 629 |  |

Waldenstrom J, Bensch S, Hasselquist D, Ostman O. A new nested polymerase chain reaction method very efficient in detecting Plasmodium and Haemoproteus infections from avian blood. J Parasitol. 2004;90: 191–194. doi:10.1645/GE-3221RN

Bensch S, Stjernman M, Hasselquist D, Ostman O, Hansson B, Westerdahl H, et al. Host specificity in avian blood parasites: a study of Plasmodium and Haemoproteus mitochondrial DNA amplified from birds. Proc R Soc B-Biol Sci. 2000;267: 1583–1589.

Petrie A. regclass: Tools for an Introductory Class in Regression and Modeling. 2017. Available: https://CRAN.R-project.org/package=regclass

R Core Team. R: A language and environment for statistical computing. Vienna, Austria: R Foundation for Statistical Computing; 2014. Available: http://www.R- project.org/

Bates D, Maechler M, Bolker BM, Walker SC. Fitting Linear Mixed-Effects Models Using lme4. J Stat Softw. 2015;67: 1–48. doi: [10.18637/jss.v067.i01](http://dx.doi.org/10.18637/jss.v067.i01)

Fox J, Weisberg S. An R Companion to Applied Regression. SAGE Publications; 2018.

Barton K. MuMIn: Multi-Model Inference. 2019. Available: https://CRAN.R- project.org/package=MuMIn

Sehgal RNM, Buermann W, Harrigan RJ, Bonneaud C, Loiseau C, Chasar A, et al. Spatially explicit predictions of blood parasites in a widely distributed African rainforest bird. Proc R Soc B-Biol Sci. 2011;278: 1025–1033. doi:10.1098/rspb.2010.1720

Cadena-Ortiz H, Mantilla JS, de Aguilar JR, Flores D, Bahamonde D, Matta NE, et al. Avian haemosporidian infections in rufous-collared sparrows in an Andean dry forest: diversity and factors related to prevalence and parasitaemia. Parasitology. 2019;146: 765–773. doi:10.1017/S0031182018002081

Garamszegi LZ. Climate change increases the risk of malaria in birds. Glob Change Biol. 2011;17: 1751–1759. doi:10.1111/j.1365-2486.2010.02346.x

Lachish S, Knowles SCL, Alves R, Wood MJ, Sheldon BC. Infection dynamics of endemic malaria in a wild bird population: parasite species-dependent drivers of spatial and temporal variation in transmission rates. J Anim Ecol. 2011;80: 1207–1216. doi:10.1111/j.1365-2656.2011.01893.x

Cosgrove CL, Wood MJ, Day KP, Sheldon BC. Seasonal variation in Plasmodium prevalence in a population of blue tits *Cyanistes caeruleus*. J Anim Ecol. 2008;77: 540– 548. doi:10.1111/j.1365-2656.2008.01370.x

Cornelius JM, Zylberberg M, Breuner CW, Gleiss AC, Hahn TP. Assessing the role of reproduction and stress in the spring emergence of haematozoan parasites in birds. J Exp Biol. 2014;217: 841–849. doi:10.1242/jeb.080697

Sol D, Jovani R, Torres J. Parasite mediated mortality and host immune response explain age-related differences in blood parasitism in birds. Oecologia. 2003;135: 542–547. doi:10.1007/s00442-003-1223-6

| 630 | 80. |
| --- | --- |
| 631 |  |
| 632 |  |
| 633 | 81. |
| 634 |  |
| 635 |  |
| 636 | 82. |
| 637 |  |
| 638 |  |
| 639 | 83. |
| 640 |  |
| 641 |  |
| 642 | 84. |
| 643 |  |
| 644 |  |
| 645 | 85. |
| 646 |  |
| 647 |  |
| 648 | 86. |
| 649 |  |
| 650 |  |
| 651 | 87. |
| 652 |  |
| 653 |  |
| 654 | 88. |
| 655 |  |
| 656 |  |
| 657 | 89. |
| 658 |  |
| 659 |  |
| 660 |  |
| 661 | 90. |
| 662 |  |
| 663 |  |
| 664 | 91. |
| 665 |  |
| 666 |  |
| 667 | 92. |
| 668 |  |
| 669 |  |
| 670 |  |

Dunn JC, Goodman SJ, Benton TG, Hamer KC. Avian blood parasite infection during the non-breeding season: an overlooked issue in declining populations? BMC Ecol. 2013;13: 30. doi:10.1186/1472-6785-13-30

Schoenle LA, Schoepf I, Weinstein NM, Moore IT, Bonier F. Higher plasma corticosterone is associated with reduced costs of infection in red-winged blackbirds. Gen Comp Endocrinol. 2018;256: 89–98. doi:10.1016/j.ygcen.2017.07.006

Garvin MC, Schoech SJ. Hormone levels and infection of Haemoproteus danilewskyi in free-ranging blue jays (*Cyanocitta cristata*). J Parasitol. 2006;92: 659–662. doi:10.1645/GE-759R.1

Isaksson C, Sepil I, Baramidze V, Sheldon BC. Explaining variance of avian malaria infection in the wild: the importance of host density, habitat, individual life-history and oxidative stress. BMC Ecol. 2013;13: 15. doi:10.1186/1472-6785-13-15

Ellis VA, Kunkel MR, Ricklefs RE. The ecology of host immune responses to chronic avian haemosporidian infection. Oecologia. 2014;176: 729–737. doi:10.1007/s00442- 014-3048-x

Arriero E, Pérez-Tris J, Ramírez A, Remacha C. Trade-off between tolerance and resistance to infections: an experimental approach with malaria parasites in a passerine bird. Oecologia. 2018;188: 1001–1010. doi:10.1007/s00442-018-4290-4

Bichet C, Cornet S, Larcombe S, Sorci G. Experimental inhibition of nitric oxide increases *Plasmodium relictum* (lineage SGS1) parasitaemia. Exp Parasitol. 2012;132: 417–423. doi:10.1016/j.exppara.2012.09.008

Graham AL, Allen JE, Read AF. Evolutionary causes and consequences of immunopathology. Annual Review of Ecology Evolution and Systematics. 2005;36: 373-397. doi: [10.1146/annurev.ecolsys.36.102003.152622](https://doi.org/10.1146/annurev.ecolsys.36.102003.152622)

Evans KJ, Hansen DS, van Rooijen N, Buckingham LA, Schofield L. Severe malarial anemia of low parasite burden in rodent models results from accelerated clearance of uninfected erythrocytes. Blood. 2006;107: 1192–1199. doi:10.1182/blood-2005-08-

3460

Price RN, Simpson JA, Nosten F, Luxemburger C, Hkirjaroen L, Kuile F ter, et al. Factors contributing to anemia after uncomplicated falciparum malaria. Am J Trop Med Hyg. 2001;65: 614–622. doi:10.4269/ajtmh.2001.65.614

Applegate JE. Population Changes in Latent Avian Malaria Infections Associated with Season and Corticosterone Treatment. J Parasitol. 1970;56: 439–443. doi:10.2307/3277599

Schoenle LA, Moore IT, Dudek AM, Garcia EB, Mays M, Haussmann MF, et al. Exogenous glucocorticoids amplify the costs of infection by reducing resistance and tolerance, but effects are mitigated by co-infection. Proc R Soc B-Biol Sci. 2019;286: 20182913. doi:10.1098/rspb.2018.2913

671 93. Cain DW, Cidlowski JA. Immune regulation by glucocorticoids. Nat Rev Immunol.

672	2017;17: 233–247. doi:10.1038/nri.2017.1

673	94. Elenkov IJ. Glucocorticoids and the Th1/Th2 Balance. Annals of the New York

674	Academy of Sciences. 2004;1024: 138–146. doi:10.1196/annals.1321.010

675	95. Martin LB. Stress and immunity in wild vertebrates: Timing is everything. Gen Comp

676	Endocrinol. 2009;163: 70–76. doi:10.1016/j.ygcen.2009.03.008

677	96. Michael Romero L. Seasonal changes in plasma glucocorticoid concentrations in free-

678	living	vertebrates. General	and	Comparative	Endocrinology. 2002;128: 1–24.

679	doi:10.1016/S0016-6480(02)00064-3

680	97. Raouf SA, Smith LC, Brown MB, Wingfield JC, Brown CR. Glucocorticoid hormone	681	levels increase with group size and parasite load in cliff swallows. Animal Behaviour. 682	2006;71: 39–48. doi:10.1016/j.anbehav.2005.03.027

683	98. Lobato E, Merino S, Moreno J, Morales J, Tomás G, Martínez- de la Puente J, et al. 684	Corticosterone metabolites in blue tit and pied flycatcher droppings: Effects of brood 685	size, ectoparasites and temperature. Hormones and Behavior. 2008;53: 295–305. 686	doi:10.1016/j.yhbeh.2007.10.010

687 99. Bosholn M, Anciães M, Gil D, Weckstein JD, Dispoto JH, Fecchio A. Individual 688	variation in feather corticosterone levels and its influence on haemosporidian infection 689	in a Neotropical bird. Ibis. 2020;162: 215–226. doi:10.1111/ibi.12709

690 100. Shutler D, Clark RG, Rutherford ST, Mullie A. Blood Parasites, Clutch Volume, and	691	Condition of Gadwalls and Mallards. Journal of Avian Biology. 1999;30: 295–301. 692	doi:10.2307/3677355

693 101. Shurulinkov P, Chakarov N, Daskalova G. Blood parasites, body condition, and wing	694	length in two subspecies of yellow wagtail (*Motacilla flava*) during migration. Parasitol 695	Res. 2012;110: 2043–2051. doi:10.1007/s00436-011-2733-5

696	102. Beldomenico PM, Begon M. Disease spread, susceptibility and infection intensity:

697	vicious circles? Trends Ecol Evol. 2010;25: 21–27. doi:10.1016/j.tree.2009.06.015

698 103. Beldomenico PM, Telfer S, Gebert S, Lukomski L, Bennett M, Begon M. Poor condition 699	and infection: a vicious circle in natural populations. Proceedings of the Royal Society 700	B: Biological Sciences. 2008;275: 1753–1759. doi:10.1098/rspb.2008.0147

701	104. Bedhomme S, Agnew P, Sidobre C, Michalakis Y. Virulence reaction norms across a

702	food gradient. Proc R Soc B-Biol Sci. 2004;271: 739–744. doi:10.1098/rspb.2003.2657

703	105. Fletcher K, Traff J, Gustafsson L. Importance of infection of haemosporidia blood	704	parasites during different life history stages for long-term reproductive fitness of collared 705	flycatchers. J Avian Biol. 2019;50: UNSP e02118. doi:10.1111/jav.02118

706	106. Svensson EI, Råberg L. Resistance and tolerance in animal enemy–victim coevolution.

707	Trends Ecol Evol. 2010;25: 267–274. doi:10.1016/j.tree.2009.12.005

708 107. Schneider DS, Ayres JS. Two ways to survive infection: what resistance and tolerance	709	can teach us about treating infectious diseases. Nat Rev Immunol. 2008;8: 889–895. 710	doi:10.1038/nri2432

711	108. Lapointe DA, Atkinson CT, Samuel MD. Ecology and conservation biology of avian

712	malaria. Ann	N	Y	Acad	Sci. 2012;1249: 211–226. doi:10.1111/j.1749-

713	6632.2011.06431.x

714 109. Peach WJ, Mallord JW, Ockendon N, Orsman CJ, Haines WG. Invertebrate prey 715	availability limits reproductive success but not breeding population size in suburban 716	House Sparrows *Passer domesticus*. Ibis. 2015;157: 601–613. doi:10.1111/ibi.12264

717 110. Freeman SN, Crick HQP. Population dynamics of House Sparrows Passer domesticus 718	breeding in Britain: an integrated analysis. HQP Crick, RA Robinson, GF Appleton, NA 719	Clark, AD Rickard. Investigation into the causes of the decline of starlings and house 720	sparrows in Great Britain BTO research report No 290. HQP Crick, RA Robinson, GF 721	Appleton, NA Clark, AD Rickard. Bristol, UK: DEFRA; 2002. pp. 193–212.

722	111. Atkinson CT, Iii C van R. Pathogenicity and epizootiology of avian haematozoa:

723	Plasmodium, Haemoproteus, and Leucocytozoon. 1991; 19–48.

724 112. Clark NJ, Clegg SM, Lima MR. A review of global diversity in avian haemosporidians 725	(Plasmodium and Haemoproteus: Haemosporida): new insights from molecular data. 726	International	Journal	for	Parasitology.	2014;44: 329–338.

727	doi:10.1016/j.ijpara.2014.01.004

728 113. Gutiérrez-López R, Puente JM la, Gangoso L, Soriguer R, Figuerola J. *Plasmodium* 729	transmission differs between mosquito species and parasite lineages. Parasitology. 730	undefined/ed; 1–7. doi:10.1017/S0031182020000062

731 114. Palinauskas V, Valkiunas G, Bolshakov CV, Bensch S. *Plasmodium relictum* (lineage 732	SGS1) and *Plasmodium ashfordi* (lineage GRW2): The effects of the co-infection on 733	experimentally	infected	passerine	birds. Exp	Parasitol. 2011;127: 527–533.

734	doi:10.1016/j.exppara.2010.10.007

735 115. Pigeault R, Cozzarolo C-S, Choquet R, Strehler M, Jenkins T, Delhaye J, et al. 736	Haemosporidian infection and co-infection affect host survival and reproduction in wild 737	populations	of	great	tits. Int	J	Parasit. 2018;48: 1079–1087.

738	doi:10.1016/j.ijpara.2018.06.007

739

# 740 Supporting information

741 S1 Table. Relationship between sex, population and capture date, and blood parasite infection
742 for both adults and juveniles.
743 S2 Table. Relationship between infection status, sex, population, and capture date, and (A)
744 physiological parameters, (B) morphological measurements and (C) condition indices for both
745 adults and juveniles.
746 S1 Fig. (A) Body mass and (B) tarsus length in relation to infection status (0 = uninfected, 1 =
747 infected) and sex in adult house sparrows.
748 S2 Fig. Body condition in relation to population and infection status in adult house sparrows.

# 749 Tables

751 Table 1. Sample size (N), prevalence, physiological and morphological values of the four studied house sparrow populations.

| Population | Population type | N  (adults/juveniles) | Blood parasite | Haematocrit  ± SD | Baseline corticosterone | Stress-induced corticosterone | Body mass | Tarsus length ± SD | Wing length | Fat score  ± SD | Muscle score |
| --- | --- | --- | --- | --- | --- | --- | --- | --- | --- | --- | --- |
|  |  |  | prevalence |  | ± SD (ng.ml^-1^) | ± SD (ng.ml^-1^) | ± SD (g) | (mm) | ± SD (mm) |  | ± SD |
| CEBC | Rural | 31 | 0.35 | 0.46 | 4.02 | 35.57 | 26.74 | 18.71 | 73.92 | 1.42 | 2.08 |
|  |  | (18/13) |  | ± 0.06 | ± 3.68 | ± 14.79 | ± 2.34 | ± 0.81 | ± 3.54 | ± 0.47 | ± 0.47 |
| Villefollet | Rural | 28 | 0.39 | 0.46 | 3.15 | 26.77 | 27.23 | 19.04 | 74.71 | 1.50 | 2.30 |
|  |  | (19/9) |  | ± 0.06 | ± 3.92 | ± 12.36 | ± 2.04 | ± 0.88 | ± 3.41 | ± 0.45 | ± 0.53 |
| Niort | Urban | 24 | 0.42 | 0.47 | 3.01 | 20.70 | 25.46 | 18.30 | 73.88 | 1.77 | 1.92 |
|  |  | (15/9) |  | ± 0.05 | ± 3.12 | ± 8.21 | ± 2.47 | ± 0.77 | ± 4.29 | ± 0.71 | ± 0.41 |
| La Rochelle | Urban | 30 | 0.40 | 0.45 | 3.06 | 29.76 | 24.60 | 17.92 | 74.71 | 1.85 | 2.17 |
|  |  | (16/14) |  | ± 0.06 | ± 3.23 | ± 13.04 | ± 1.92 | ± 0.81 | ± 3.41 | ± 0.56 | ± 0.53 |

752 SD: Standard deviation
754 Table 2. Results from the GLMs of the blood *Plasmodium* infection status (n = 113) and sex, population and capture date in house sparrows obtained without non-significant interaction terms. Significant effects (p < 0.05) are highlighted in bold. *R^2^* explained variance is shown.

| Dependent variable  Parameter | Infection status  Estimate ± SE | z-value | p-value |
| --- | --- | --- | --- |
| Intercept | -0.21 ± 0.53 | -0.40 | 0.690 |
| Age (juvenile) | **-1.34 ± 0.49** | **-2.73** | **0.006** |
| Sex (male) | 0.75 ± 0.42 | 1.76 | 0.079 |
| Population (Villefollet) | -0.35 ± 0.60 | -0.59 | 0.555 |
| Population (Niort) | -0.04 ± 0.61 | -0.06 | 0.953 |
| Population (CEBC) | -0.14 ± 0.59 | -0.24 | 0.813 |
| Capture date | **0.66 ± 0.25** | **2.58** | **0.010** |

R^2^	0.15

755 Table 3. Relationship between blood parasite infection status, age, sex, and population, and (A) physiological parameters, (B) morphological
756 measurements and (C) condition indices in house sparrows (n = 113) obtained from Linear Models without non-significant interaction terms.
757 Significant effects (p < 0.05) are in bold. ‘-‘ means a parameter was not fitted to the model and ‘:’ represents interactions.
    A. **Physiological parameters**

Dependent variable	Haematocrit	Baseline corticosterone	Stress-induced corticosterone

| Parameter | Estimate ± SE | t-value | p-value | Estimate ± SE | t-value | p-value | Estimate ± SE | t-value | p-value |
| --- | --- | --- | --- | --- | --- | --- | --- | --- | --- |
| Intercept | **0.46 ± 0.01** | **34.46** | **<0.001** | **4.53 ± 0.82** | **5.51** | **<0.001** | **37.27 ± 2.27** | **16.41** | **<0.001** |
| Infection status (infected) | -0.01 ± 0.01 | -0.59 | 0.558 | -0.16 ± 0.63 | -0.25 | 0.803 | -2.09 ± 1.75 | -1.20 | 0.233 |
| Age (juvenile) | **-0.03 ± 0.01** | **-2.31** | **0.023** | **-1.92 ± 0.694** | **-2.77** | **0.007** | **-8.98 ± 2.07** | **-4.35** | **<0.001** |
| Sex (male) | 0.00 ± 0.01 | 0.50 | 0.960 | -0.84 ± 0.60 | -1.39 | 0.167 | **-3.68 ± 1.68** | **-2.20** | **0.030** |
| Population (Villefollet) | 0.02 ± 0.01 | 1.29 | 0.201 | -0.11 ± 0.84 | -0.13 | 0.893 | **-5.01 ± 2.31** | **-2.16** | **0.033** |
| Population (Niort) | 0.03 ± 0.01 | 1.93 | 0.056 | -0.45 ± 0.88 | -0.52 | 0.605 | **-11.76 ± 2.41** | **-4.88** | **<0.001** |
| Population (CEBC) | 0.01 ± 0.01 | 0.75 | 0.455 | 0.28 ± 0.88 | 0.31 | 0.755 | 0.89 ± 2.44 | 0.36 | 0.716 |
| Capture date | **-0.02 ± 0.01** | **-3.41** | **0.001** | **-1.73 ± 0.55** | **-3.12** | **0.002** | **-9.50 ± 1.59** | **-5.98** | **<0.001** |
| Population (Villefollet):capture date | - | - | - | 0.27 ± 0.79 | 0.34 | 0.733 | 0.73 ± 2.18 | 0.33 | 0.740 |
| Population (Niort):capture date | - | - | - | **2.54 ± 0.85** | **2.98** | **0.004** | **10.18 ± 2.37** | **4.31** | **<0.001** |
| Population (CEBC):capture date | - | - | - | 0.53 ± 0.93 | 0.57 | 0.568 | -1.10 ± 2.58 | -0.42 | 0.672 |
| Age (juvenile):capture date | - | - | - | - | - | - | **5.06 ± 2.23** | **2.72** | **0.025** |

R^2^	0.25	0.31	0.64

| **B. Morphological parameters** |  | | | | | | | | |
| --- | --- | --- | --- | --- | --- | --- | --- | --- | --- |
| Dependent variable | Body mass |  |  | Tarsus length |  |  | Wing length |  |  |
| Parameter | Estimate ± SE | t-value | p-value | Estimate ± SE | t-value | p-value | Estimate ± SE | t-value | p-value |
| Intercept | 27.54 ± 0.52 | 53.11 | <0.001 | **18.87 ± 0.21** | **89.13** | **<0.001** | **74.20 ± 0.58** | **128.04** | **<0.001** |
| Infection status (infected) | -0.49 ± 0.64 | -0.77 | 0.444 | -0.49 ± 0.25 | -1.96 | 0.053 | 0.64 ± 0.45 | 1.40 | 0.164 |
| Age (juvenile) | **-3.34 ± 0.66** | **-5.06** | **<0.001** | **-1.03 ± 0.25** | **-4.15** | **<0.001** | **-4.91 ± 0.50** | **-9.76** | **<0.001** |
| Sex (male) | -0.64 ± 0.59 | -1.09 | 0.280 | -0.34 ± 0.24 | -1.44 | 0.154 | **2.38 ± 0.43** | **5.49** | **<0.001** |
| Population (Villefollet) | 0.54 ± 0.51 | 1.08 | 0.283 | **0.55 ± 0.20** | **2.70** | **0.008** | 0.42 ± 0.60 | 0.71 | 0.482 |
| Population (Niort) | **-1.06 ± 0.52** | **-2.05** | **0.043** | -0.09 ± 0.21 | -0.42 | 0.675 | 0.30 ± 0.63 | 0.48 | 0.636 |
| Population (CEBC) | **-1.93 ± 0.50** | **-3.84** | **<0.001** | **-0.62 ± 0.21** | **-2.97** | **0.004** | -0.04 ± 0.61 | -0.06 | 0.952 |

| Capture date | **-0.55 ± 0.23** | **-2.43** | **0.017** | **-0.39 ± 0.13** | **-3.01** | **0.003** | -0.37 ± 0.26 | -1.41 | 0.161 |
| --- | --- | --- | --- | --- | --- | --- | --- | --- | --- |
| Infection status (infected): age (juvenile) | 1.77 ± 1.18 | 1.50 | 0.136 | **1.37 ± 0.46** | **2.98** | **0.004** | - | - | - |
| Infection status (infected):sex (male) | 1.52 ± 0.88 | 1.73 | 0.087 | **0.80 ± 0.35** | **2.31** | **0.023** | - | - | - |
| Sex (male): age (juvenile) | 1.45 ± 0.95 | 1.54 | 0.128 | 0.71 ± 0.36 | 1.96 | 0.053 | - | - | - |
| Age (juvenile):capture date | **1.67 ± 0.51** | **3.28** | **0.001** | - | - | - | - | - | - |
| Population (Villefollet):capture date | - | - | - | 0.16 ± 0.19 | 0.88 | 0.384 | - | - | - |
| Population (Niort):capture date | - | - | - | 0.11 ± 0.20 | 0.53 | 0.597 | - | - | - |
| Population (CEBC):capture date | - | - | - | **0.68 ± 0.22** | **3.09** | **0.003** | - | - | - |
| Infection status (infected):age (juvenile):sex (male) | **-3.37 ± 1.57** | **-2.15** | **0.034** | **-1.31 ± 0.61** | **-2.13** | **0.036** | - | - | - |

R^2^	0.49	0.46	0.65

| **C. Condition indexes** |  | | | | | | | | |
| --- | --- | --- | --- | --- | --- | --- | --- | --- | --- |
| Dependent variable | Fat score |  |  | Muscle score |  |  | Body condition |  |  |
| Parameter | Estimate ± SE | Z-value | p-value | Estimate ± SE | Z-value | p-value | Estimate ± SE | t-value | p-value |
| Intercept | **0.96 ± 0.15** | **6.33** | **<0.001** | **1.41 ± 0.13** | **11.01** | **<0.001** | -0.10 ± 0.17 | -0.56 | 0.574 |
| Infection status (infected) | -0.06 ± 0.11 | -0.49 | 0.625 | -0.05 ± 0.10 | -0.55 | 0.582 | -0.12 ± 0.16 | -0.75 | 0.452 |
| Age (juvenile) | **0.26 ± 0.12** | **2.10** | **0.036** | -0.08 ± 0.11 | -0.71 | 0.480 | -0.01 ± 0.17 | -0.08 | 0.938 |
| Sex (male) | 0.02 ± 0.11 | 0.16 | 0.877 | 0.06 ± 0.09 | 0.65 | 0.514 | 0.06 ± 0.12 | 0.48 | 0.634 |
| Population (Villefollet) | 0.05 ± 0.16 | 0.31 | 0.755 | 0.13 ± 0.13 | 0.99 | 0.322 | 0.28 ± 0.17 | 1.67 | 0.098 |
| Population (Niort) | 0.20 ± 0.16 | 1.29 | 0.199 | -0.04 ± 0.14 | -0.25 | 0.800 | 0.05 ± 0.18 | 0.31 | 0.759 |
| Population (CEBC) | 0.20 ± 0.15 | 1.31 | 0.190 | 0.11 ± 0.13 | 0.86 | 0.389 | -0.10 ± 0.17 | -0.55 | 0.586 |
| Capture date | 0.07 ± 0.07 | 1.03 | 0.304 | -0.07 ± 0.06 | -1.18 | 0.239 | **-0.17 ± 0.07** | **-2.27** | **0.026** |
| Infection status (infected):age (juvenile) | - | - | - | - | - | - | **0.53 ± 0.26** | **2.06** | **0.042** |

R^2^	0.14	0.05	0.13

758

# 759 Figure captions

761 Fig 1. Location of the four house sparrow populations sampled in this study. Brown and green
762 dots represent urban and rural sites, respectively.
764 Fig 2. Proportion of infected individuals in relation to (A) population and (B) capture date in
765 adults and juvenile house sparrows. (A) Dots with error bars indicate the means (± SE). Black
766 dots correspond to the adults and grey dots to the juveniles. (B) Dots represent the infection
767 status of the individuals. The black and grey solid lines with standard errors (grey areas)
768 represent the model predictions for adults and juveniles, respectively.
770 Fig 3. (A) Haematocrit, (B) baseline corticosterone level, (C) stress-induced corticosterone
771 level, (D) body mass, (E) tarsus length, (F) wing length and (G) body condition in relation to
772 infection status (0 = uninfected, 1 = infected) and age. Dots represent the raw data. Grey
773 boxplots represent adults and white boxplots represent juveniles.

Figure 1	[Click here to access/download;Figure;Figure1.pdf](https://www.editorialmanager.com/pone/download.aspx?id=26411077&amp;guid=082c3e3c-5a4d-4347-b3d5-f0dcbb4fd7c6&amp;scheme=1)

Figure 2	[Click here to access/download;Figure;Figure2.pdf](https://www.editorialmanager.com/pone/download.aspx?id=26411078&amp;guid=79b4d0a3-1d46-4249-b1a1-37a083fbdf06&amp;scheme=1)

A	B

Supporting Information

[Click here to access/download](https://www.editorialmanager.com/pone/download.aspx?id=26411093&amp;guid=1f0a06ea-42b2-46ca-8ae4-4fef3d2283ec&amp;scheme=1)

[Supporting Information SI_150420.docx](https://www.editorialmanager.com/pone/download.aspx?id=26411093&amp;guid=1f0a06ea-42b2-46ca-8ae4-4fef3d2283ec&amp;scheme=1)
