# Supplementary material for: Physiological and morphological correlates of blood parasite infection in urban and non-urban house sparrow populations
Source: PLoS One. 2020 Aug 19;15(8):e0237170. doi: 10.1371/journal.pone.0237170 (PMC7437892; doi:10.1371/journal.pone.0237170)
Supplement: Attachment — Submitted filename: reply_malaria-physio_030720.docx [file pone.0237170.s006.docx]

**PONE-D-20-04379R1**

**“Physiological and morphological correlates of blood parasite infection in urban and non-urban house sparrow populations”**

***- Our responses to comments are provided in bold and italic font. We also added a version of our manuscript where changes from the previous version appear highlighted in blue -***

Dear Dr. Bichet,

Thank you for submitting your manuscript to PLOS ONE. After careful consideration, we feel that it has merit but does not fully meet PLOS ONE’s publication criteria as it currently stands. Therefore, we invite you to submit a revised version of the manuscript that addresses the points raised during the review process.

The reviewers and I came to the consensus that the manuscript still deserves attention. Two reviewers point out that the authors did not respond to the questions raised adequately. I strongly agree with them and emphasize that the authors must dedicate to answer the points raised with the utmost precision and to make all those reconsiderations in the manuscript to be submitted.

***> We are very grateful to the associated editor and to the reviewers that our study still deserves attention. We are surprised to read that we did not answer some questions adequately. We are always happy and grateful to follow all the comments we obtained, since we really believe that the peer-review process is the best way to improve every studies. We did our best to include these suggestions. Some of them were very interesting and we addressed them. However, we are not convinced that others were relevant in the context of our study and we explained why in the previous round of review (it may be due to some misunderstanding and some repeated typos in the review of the reviewers, ex: reviewer 1 asked to correct for sample size, while he/she meant to correct by sample site, reviewer saying we did not take into account one previous request we cannot find in his/her first report). We now better understand what the reviewers meant and we have modified the manuscript according to most of the comments. For a few of them, we clearly justify why we have not processed with the suggested modifications (see our detailed answers below).***

We would appreciate receiving your revised manuscript by Jun 20 2020 11:59PM. When you are ready to submit your revision, log on to <https://www.editorialmanager.com/pone/> and select the 'Submissions Needing Revision' folder to locate your manuscript file.

If you would like to make changes to your financial disclosure, please include your updated statement in your cover letter.

To enhance the reproducibility of your results, we recommend that if applicable you deposit your laboratory protocols in [protocols.io](http://protocols.io/), where a protocol can be assigned its own identifier (DOI) such that it can be cited independently in the future. For instructions see: <http://journals.plos.org/plosone/s/submission-guidelines#loc-laboratory-protocols>

Please include the following items when submitting your revised manuscript:

- A rebuttal letter that responds to each point raised by the academic editor and reviewer(s). This letter should be uploaded as separate file and labeled 'Response to Reviewers'.
- A marked-up copy of your manuscript that highlights changes made to the original version. This file should be uploaded as separate file and labeled 'Revised Manuscript with Track Changes'.
- An unmarked version of your revised paper without tracked changes. This file should be uploaded as separate file and labeled 'Manuscript'.

Please note while forming your response, if your article is accepted, you may have the opportunity to make the peer review history publicly available. The record will include editor decision letters (with reviews) and your responses to reviewer comments. If eligible, we will contact you to opt in or out.

We look forward to receiving your revised manuscript.

Kind regards,

Érika Martins Braga, Ph.D.

Academic Editor

PLOS ONE

**Reviewers' comments:**

**Reviewer #1**

In this new version, authors have been improved the contents of the manuscript, addressing some of the queries proposed by reviews, although I think most of the ‘content’ changes are still missing. The authors have addressed the majority of the queries proposed with a too vague and not entirely conclusive tone. Also, I have noticed that authors avoided to give concrete answers to important and fundamental questions from a data analysis point of view.

***> We are very sorry we gave this impression to the reviewer. We are always happy and grateful to follow all the comments we obtained, since we really believe that the peer-review process is the best way to improve every studies. We did our best to include these suggestions. Some of them were very interesting and we addressed them. However, we are not convinced that others were relevant in the context of our study and we explained why in the previous round of review (it may be due to some misunderstanding and some repeated typos in the review of the reviewer, ex: the reviewer asked to correct for sample size, while he/she meant to correct by sample site). We now better understand what the reviewer meant and we have modified the manuscript according to most of the comments. For a few of them, we explain in details why we have not processed with the suggested modifications (see our detailed answers below).***

The main problem I still see, is the lack of a complete model were all independent variables (physiological, morphological, and condition indices) are analysed at the same time by using infection status as dependent. Authors perform several little GLMs were they test different variables with infectious status but the overall effect is missing. Which are the most important variables? Which variable are driving infection in sparrows? I suggest this because the reader too often gets lost among so many non-significant results, tables, fragmented figures, supplementary information with significant results, sometimes some of them nor discussed in the manuscript.

I can suggest a general model with all the independent variables together in a AIC selection in order to have a more complete vision of the reality.

***> We are deeply sorry, but we have carefully read again the first round of reviews and we could not see where the reviewer asked for these full models in his/her first review. That is why we did not reply to this comment. We are also not convinced by this request because the physiological, morphological and condition variables were never the independent variables (see lines 180-184) in our models. Our goal was to understand whether these variables could be affected by blood parasite infections and not the opposite (all variables were entered as dependent variables, i.e. variables to be explained). We do not understand why the referee wants to have them as independent (explanatory) variables because it does not really make sense to test whether these variables could determine if a bird will be infected of not. The goal was to understand the impact of infection on organismal systems.***

***Regarding the variables driving the infection, we have already conducted this model, including together all the relevant variables (population, capture date, age, sex, and now urbanisation score according to reviewer 1 and reviewer 2’s requests) which may affect the infection status (see lines 172-179). Therefore, we believe that we have already run the biologically-relevant full models to explain the infection status. We are sorry if we have missed something but we honestly do not think that the suggested models are relevant for this study.***

***However, to simplify our main message, we have shortened and clarified the result section (for instance lines 217, 231, 239, 244, 257, 240-242) and we have presented the results for adults and juveniles in the Tables 2 and 3 in the main manuscript and not anymore in the supplemental tables (Tables S1 and S2 deleted from the supplemental and added in Tables 2 and 3, respectively). But, to follow the reviewer 2’s request, we also have to provide more details regarding some significant interactions, in the result section, which lengthens this section a little bit (lines 231-235, 240-242, 250-258).***

In this model, authors should include a categorical variable of the sampling site (no size), as I previous commented them in the first review (but that authors said did not understand).

***> We apologize we did not make this modification. In the first review, the reviewer requested to “try to control for the sampling size (N) as another covariate in your models. This could be a better option in order to control for the limitation of the small sampling size” and that “authors should control for the sampling site N in each population”. For us it was clear that the reviewer asked to control for sample SIZE and that the second comment contained a typo (site instead of size, because the “N” was mentioned in both, and N is usually used to refer to sample size). Regarding the current comment about sample SITE: all our models already include the sample site as a categorical variable since our first submission. This variable is called “population” (lines 177, 188) in our manuscript and all our tables (Tables 2 and 3). Therefore, we do not know how to satisfy the referee regarding this point: if the reviewer is talking about sampling SITE, this variable is already present in all our models. However, please note that according to next comments and reviewer 2’s request, we have modified our models and the population (or sampling site) is now added as a random factor in all our models (lines 177 and 188).***

Results about no differences between habitat category (urban/rural), should be also taken with care. As I suggest previously, habitat category (urban/rural) or sampling site (the four localities, as categorical), should have been a variable took into account in the other GLMs, and used as random factor (glmer function in R) when authors want to get pattern about independent variables that could be driving infection in birds, disengaging from environmental patterns.

***> According to this comment, we have included the urbanization score as an explanatory variable and the sampling sites/populations as a random factor in our model (lines 113-114, 176 and 187, Table 1). This does not modify our results or our interpretations (Tables 2, 3, result and discussion sections).***

Also, the manuscript has still lots of minor weaknesses that were pointed out in the text I attached.
***> We thank the reviewer for the attached document and we have now corrected all the points mentioned in it (e.g. lines 41, 113, 157, 175, 196, 366).***

***Regarding the specific comment line 120: It is true that banding the bird is usually the first task after capture, but in this case, blood sampling has to be done within 3 minutes after capture to be able to access baseline corticosterone (lines 121-122), and therefore, blood sampling was definitely the first thing to do, before banding.***

***Regarding the specific comment line 272, “Authors should include the previous ‘Urbanization Score’ they have in order to control for this effect and solve this problem”. As suggested, we have added these models in our manuscript (lines 112-113, 176 and 187) and we have modified the manuscript accordingly (methods, results and discussion). It does not change our results and there is no clear link between urbanization and the probability of infection (see also our answer to the previous related comment).***

**Reviewer #2:**

Authors correctly clarify the most part of the issues raised in the first major revision, but they still have considerable points that were not solved in the first time, or needs to be clarify even more (previous point 3 to 5). In detail:

3) Authors should have standardized the use about “infection status or probability” terms. They have corrected it in the most part of the manuscript, but in the first part of results (lines 201-210), they still refer to “The number of infected individuals” which is not correct as in the table cited they use “infection status”.

***> We thank the reviewer for this suggestion and we now refer to “probability of being infected” (lines 211, 212, 214 and 216). We hope that this term will avoid any further confusion.***

Moreover, in the figure 2B, they labelled the Y axis as “infection status” that is supposed to be a binary variable, but the axis has intermediate value. Why? Please, correct this ‘again’ and make sure that you standardize it all along the manuscript, including figures, tables, and Supplementary Information.

***> We added the intermediate values to help to visualize predictions obtained from the model. For example, if you want to know the probability of infection for an adult bird caught at day 210, the model gives you a probability of infection around 0.5. We thought it was useful and informative, but we now deleted it and only keep the ticks 1 and 0 in the Figure 2B. We also corrected the legend of the Table 2 by replacing “blood parasite infection” by “infection status” (lines 749, 753, 764).***

4) Authors were asked to consider the results from Jiménez-Peñuela et al. 2019 in their study and even they include it in line 62 and 326, they did not consider it in their third hypothesis proposed in the discussion, that is the same hypothesis proposed in this work as I said in the first review. Moreover, in lines 66-68 they made an inappropriate statement “has never been examined in house sparrow”, but during all the introduction they are referring to works that already have evaluated similar variables in house sparrows, including in the suggested reference. The same occur with the statement on lines 277-278, where I suggested softening the tone in minor comments. Please, correct any similar statements along all the manuscript ‘again’ and make sure to check what other authors have recently did in the same topic, especially the ones that you are referring along your work.

***> We apologize for this mistake. We have now included this reference when explaining the third hypothesis of the discussion (line 362). We have also removed the terminology “has never been examined in house sparrows” from the manuscript and we have made sure to refer to previous works (lines 67-68)*.**

5) Authors were asked to include either a qualitative or quantitative variable evaluating the urbanization of their population, due to as I said, including population ID in the statistical analysis only indicates if there was any difference between populations, but it did not compare urban and non-urban ones. Authors are claiming through all the manuscript that they compare urban and non-urban population (title, keywords, introduction, discussion…) but their answer to this request was: “The influence of urbanization on physiological parameter is not the topic of our study and we added population ID to control for population differences in physiological and morphological parameters already found in the previous study (Meilliere et al 2015 PlosOne 10(8))”.

I don't entirely agree with that answer and I can only comprehend two options: 1) if you do not include any variable that evaluate urbanization because it is not the topic of your work, then eliminate all the related information about urbanization from the text because you are confounding the lector about the topic of your work. 2) if on the contrary, you decide to keep going with the urbanization variable, please, include it correctly in your analysis either with a qualitative or quantitative variable, due to in the previous version of the manuscript authors claimed to already have this variable from a previous study. Besides, this will allow the authors clarify statements like did in lines 272-274 not depending in other studies, and if it is the case, they could discuss why they obtain different results for the same populations.

***> We agree and we have now added the urbanization score as a quantitative variable to take into consideration the level of urbanization (urbanisation score, lines 113-114) on our data (lines 176 and 187). We also now explicitly mention that the impact of urbanization on these variables has been previously published with the same data set and that we specifically look at the effect of malaria infection on these variables in different populations (along an urbanization gradient) (lines 292-295 and 324-327).***

Apart from old issues, there are some new points than authors need to correct in the current version of the manuscript:

A) Authors propose in the introduction two main objectives (lines 70-72 and 78-81) that did not correspond with the ones analysed in the statistical analyses (line 165 and 173) and results. Please, reformulate correctly them.

***> We have modified accordingly lines 72-74 and 81***.

B) Authors include references to supplementary material through all the results section, even though if they correspond to the principal results of their study. Thus, these parts should not be reported at supplementary but at principal tables. If the tables are too big, then prioritize the significant results or the most explicative and the others put it on supplementary material. The main results should be clarified along the manuscript with figures and tables and lectures have to get all the information with that, and only check supplementary material for extra information.

***> Accordingly, we have shortened and restructured the results section to be clearer (for instance lines 217, 231, 239, 244, 257, 240-242). Moreover, we now present the results for adults and juveniles in the Tables 2 and 3 in the main manuscript and not anymore in the supplemental (Tables S1 and S2 deleted from the supplemental and added in Tables 2 and 3, respectively). We hope that this will satisfactorily answer to the reviewer’s comment.***

C) There is no Figure 3 in the new version of the manuscript, please be aware that all the information needed is included.

***> We apologize for this mistake. Strangely, the Figure 3 was included in the pdf we received right after our resubmission. We definitely had a Figure 3 and we are making sure it is included in this new submission.***

D) Significant interactions should be more clarified and explained in the results: e.g. lines 224-227, 229-231 and 249-251.

***> According to this comment, we have modified the results to explain better the most relevant significant interactions (lines 231-235, 240-242 and 250-258).***

E) Fat and muscle score variables were not explained in the results, but they were statistical significant results with fat in juveniles. Why? Please, add and discuss them.

***> Fat and muscle scores were both not correlated with infection status in either adults or juveniles (see Table 3C), that is why there are not discussed (the aim of the analysis was to test if infection status could explain fat/muscle scores). We only mentioned that “Infection status was not associated with sparrow condition (body condition, fat, and muscle scores, Table 3c)” (lines 248-259). The fact that fat score differed between juveniles and adults (Table 3C) is not the purpose of the present study focusing on blood parasite infections and physiological, morphological and condition consequences. In these models, “age” was added as a covariate to control for expected physiological, morphological and condition differences between adults and juveniles. We have added a sentence (lines 324-327) stating that these differences were already found in a previous study using the same data set (and aiming to understand the effect of urbanization on these parameters).***

F) The text is referring to figures that do not have the correct information e.g. line 227, sex is not include in figure 3; line 244-245 juvenile size is not include in figure 2b.

***> Thank you. This is corrected (lines 235, 245).***

G) During the discussion section, some references are missing (lines 285-286, 321-322) and others are included but not explained in line 268, 295, 297 and specially in 321.

***> We included references accordingly (lines 286, 335), in a limited number, to still fit with the first round of comments stating that we had too many references, and we explained the references (lines 267-268, 298-300, 302, 330-334).***

H) The sentence in lines 161-162 should be included in results, not in materials and methods.

***> We understand why the reviewer would like this sentence to be moved to the results. We would prefer to keep it here, otherwise it will make some confusion about which parasite is taking into account to determine the infection status used in all the analyses. If the reviewer still thinks that it should be moved to the results after reading these explanations, we will be happy to do it.***

**Reviewer #3:**

I have only a few minor suggestions that should be addressed in this manuscript before publication.

Line 51 – Places with birds presenting higher parasitemia had lower bird survival. Please correct this sentence because parasitemia per se is not the reason for the population decline.

***> Thank you. This is now corrected (line 51).***

Line 117 – keep this sentence in the past tense.

***> Corrected (line 122).***

Line 121 – What does “experimenter” means? Do authors want to say something like “experienced researcher”? The same in line 129.

***> “Experimenter” means the person who made the measurements. Nothing related with the level of experience. We checked with a native speaker and this term seems appropriate in our respect.***

Line 180 – Change “injection” to “infection”.

***> Modified (line 189).***

Line 302 – Change to “thus are not sampled”.

***> Modified (line 308).***
