# Supplementary material for: Physiological and morphological correlates of blood parasite infection in urban and non-urban house sparrow populations
Source: PLoS One. 2020 Aug 19;15(8):e0237170. doi: 10.1371/journal.pone.0237170 (PMC7437892; doi:10.1371/journal.pone.0237170)
Supplement: Attachment — Submitted filename: reply_malaria-physio_170720.docx [file pone.0237170.s007.docx]

**PONE-D-20-04379R2**

**“Physiological and morphological correlates of blood parasite infection in urban and non-urban house sparrow populations”**

***- Our responses to comments are provided in bold and italic font. We also added a version of our manuscript where changes from the previous version appear highlighted in blue -***

Dear Dr. Bichet,

Thank you for submitting your manuscript to PLOS ONE. After careful consideration, we feel that it has merit but does not fully meet PLOS ONE’s publication criteria as it currently stands. Therefore, we invite you to submit a revised version of the manuscript that addresses the points raised during the review process.

==============================
The authors addressed the main concerns from the reviews. However, your revised manuscript still deserves attention. Please, provide, point-to-point responses according to the comments made the Reviewer 21 in the new version of your manuscript. 
==============================

***> We would like to thank the associate editor and the two reviewers for their comments. We replied point by point to them and we hope that this revised version will provide entire satisfaction.***

Please submit your revised manuscript by Aug 31 2020 11:59PM. If you will need more time than this to complete your revisions, please reply to this message or contact the journal office at [plosone@plos.org](mailto:plosone@plos.org). When you're ready to submit your revision, log on to <https://www.editorialmanager.com/pone/> and select the 'Submissions Needing Revision' folder to locate your manuscript file.

Please include the following items when submitting your revised manuscript:

- A rebuttal letter that responds to each point raised by the academic editor and reviewer(s). You should upload this letter as a separate file labeled 'Response to Reviewers'.
- A marked-up copy of your manuscript that highlights changes made to the original version. You should upload this as a separate file labeled 'Revised Manuscript with Track Changes'.
- An unmarked version of your revised paper without tracked changes. You should upload this as a separate file labeled 'Manuscript'.

If you would like to make changes to your financial disclosure, please include your updated statement in your cover letter. Guidelines for resubmitting your figure files are available below the reviewer comments at the end of this letter.

If applicable, we recommend that you deposit your laboratory protocols in [protocols.io](http://protocols.io/) to enhance the reproducibility of your results. Protocols.io assigns your protocol its own identifier (DOI) so that it can be cited independently in the future. For instructions see: <http://journals.plos.org/plosone/s/submission-guidelines#loc-laboratory-protocols>

We look forward to receiving your revised manuscript.

Kind regards,

Érika Martins Braga, Ph.D.

Academic Editor

PLOS ONE

[Note: HTML markup is below. Please do not edit.]

Reviewers' comments:

Reviewer #1: In this new version, authors have been considerably improved the contents of the manuscript, addressing almost all the queries proposed by reviewers. The statistical analyses have improve considerably. Though, the manuscript has few minor faults that were pointed out in the text I attached.

***> We would like to thank the reviewer for saying that our manuscript is considerably improved. We also thank the reviewer for taking the time to edit our manuscript to correct the minor remaining mistakes we had. We corrected that (e.g. lines 49, 60, 87, 274, 280, 308, 430, 608, 725). We also corrected the Tables 2 and 3 by giving three decimals to all values.***

Reviewer #2: Authors clarify correctly and solve the issues presented in the first and second major revisions. Even though the main limitations of the study that was the number of individuals use and genera identity of the parasite is still present due to it is not possible to be solved, the manuscript has improved.

> ***We would like to thank the reviewer for thinking the manuscript has improved and that we clarified and solved the issues she/he pointed out.***

I would recommend assessing some small points:

Line 122: “The blood collected was also to be used”?? maybe they should delete “to be”.

***> Deleted (line 123).***

Lines 196-197: Adult and juvenile information is no longer in supplementary information. They should correct this reference.

***> Thank you. It is corrected (line 196).***

Lines 203-204: If they say that interactions with a p-value higher than 0.05 were removed from all the models, why in Table 3 B with the analyses of body mass in all birds they keep the interaction infection status-age, infection status-sex and sex-age? Please correct this.

***> We apologize, but we cannot access this request. If the two-way interactions are not significant, the three-way interaction was significant, and therefore, the associated two-way interaction as well as the single terms have to remain in the model.***

Lines 233 and 242: Which are these post-hoc comparison? Please, specify the function used in the statistical analyses.

***> We added this information (lines 204-206).***
